# Room-Temperature Creep Behavior and Activation Volume of Dislocation Nucleation in a LiTaO_3_ Single Crystal by Nanoindentation

**DOI:** 10.3390/ma12101683

**Published:** 2019-05-23

**Authors:** Yi Ma, Xianwei Huang, Yuxuan Song, Wei Hang, Taihua Zhang

**Affiliations:** 1 College of Mechanical Engineering, Zhejiang University of Technology, Hangzhou 310014, China; may@zjut.edu.cn (Y.M.); huangxw@zjut.edu.cn (X.H.); songyux@zjut.edu.cn (Y.S.); 2 Key Laboratory of Special Purpose Equipment and Advanced Manufacturing Technology Ministry of Education, Zhejiang University of Technology, Hangzhou 310027, China; 3 Institute of Solid Mechanics, Beihang University, Beijing 100191, China

**Keywords:** lithium tantalite, nanoindentation, creep, orientation effect, strain rate sensitivity, activation volume

## Abstract

The crystal orientation effect on mechanical heterogeneity of LiTaO_3_ single crystals is well known, whilst the time-dependent plastic behavior, i.e., creep is still short of understanding. Relying on nanoindentation technology, we systematically studied room-temperature creep flows at various holding depths (100 nm to 1100 nm) in three typical orientations namely the X-112°, Y-36° and Y-42° planes. Creep resistance was much stronger in the X-112° plane than the others. In the meanwhile, creep features were similar in the Y-36° and Y-42° planes. The orientation effect on creep deformation was consistent with that on hardness. The nanoindentation length scale played an important role in creep deformation that creep strains were gradually decreased with the holding depth in all the planes. Based on strain rate sensitivity and yield stress, the activation volumes of dislocation nucleation were computed at various nanoindentation depths. The activation volumes ranged from 5 Å^3^ to 23 Å^3^ for the Y-36° and Y-42° planes, indicating that a point-like defect could be the source of plastic initiation. In the X-112° plane, the activation volume was between 6 Å^3^ and 83 Å^3^. Cooperative migration of several atoms could also be the mechanism of dislocation activation at deep nanoindentation.

## 1. Introduction

A lithium tantalite (LiTaO_3_) single crystal is a relatively new synthetic piezoelectric material, which has been widely adopted in the commercial laser and communication fields due to its excellent optical and electrical properties [1]. In particular, the development of LiTaO_3_ single crystals has greatly enhanced as an outstanding candidate for surface acoustic wave (SAW) device for its low acoustic loss [2]. The excellent surface qualities, such as extremely low roughness and good flatness, are required for successful application of LiTaO_3_ single crystals. Relying on ultra-precision machining technology, the surface with nanometer-scale roughness could be attained on LiTaO_3_. In the meanwhile, the mechanical properties and deformation mechanism at the nanoscale in a LiTaO_3_ single crystal have attracted numerous attention in order to promote the grinding and polishing efficiency [3,4]. Due to the unique atomic arrangement, the orientation effect on mechanical heterogeneity in a LiTaO_3_ single crystal is also on the cutting edge of mechanical investigation [5,6,7]. 

Creep is a time-dependent plastic deformation, which is vital to the service life of engineering materials [8]. Relying on nanoindentation technology, creep behaviors can be studied in a small region, ignoring the limitation of required standard size in conventional creep testing [9]. Moreover, the subtle creep flow could be recorded accurately due to the sub-nanometer resolution. Even though thermal drift is unavoidable, nanoindentation has been largely used to detect creep behaviors of metals and alloys in recent years [10,11,12,13,14]. For functional single crystal materials such as Si and LiTaO_3_, brittleness greatly hinders their commercial applications and development. Due to the poor processability, creep resistances of these single crystals have been scarcely investigated hitherto and the creep mechanism is not well understood. With this in mind, we aimed to reveal creep features in the three typical cleavage planes ($1\bar{1}02$), ($\bar{1}012$) and ($01\bar{1}2$) of a LiTaO_3_ single crystal, i.e., X-112°, Y-36° and Y-42°. The damage layer generated by polishing was unavoidable, which influenced the mechanical properties at the surface and/or sub-surface. Therefore, it was necessary to study the nanoindentation size effect on creep behaviors. In order to minimize thermal drift influence, creep experiments were conducted at room temperature. 

## 2. Materials and Methods 

The commercial LiTaO_3_ single crystal wafers were carefully polished at three typical orientations, respectively. Diamond abrasive at an average size of 2 µm (8000#) and a polyurethane polish pad were adopted on the Nanopoli-100. After 45 min of polishing, LiTaO_3_ samples were carefully cleaned in anhydrous alcohol by ultrasonic cleaning. The thickness and radius of the wafer were 0.25 mm and 1 inch. The surface morphologies at the center were detected by optical profiler (Zygo Newview 5022, Berwyn, PA, USA) and roughness could thus be measured. Creep testing was conducted on Agilent Nano Indenter G200 (Santa Clara, CA, USA) at a constant temperature of 20 °C with a standard Berkovich indenter. The elastic modulus and hardness of three cleavage planes were detected upon continuous stiffness module (CSM) with a constant strain rate of 0.05 s^−1^. The constant-load holding mode was adopted to explore the time-dependent plastic deformation, during which the displacement of the indenter into the surface at a prescribed load could be continuously recorded. The indenter was held for 500 s at different peak loads 5 mN, 20 mN, 50 mN, 100 mN and 250 mN. The loading rate was fixed, equal to 0.5 mN/s. The creep tests were launched until thermal drift reduced below 0.02 nm/s. Meanwhile, drift correction which was calibrated at 10% of the maximum load during the unloading process was strictly performed. To ensure the reliability of creep results, more than twenty nanoindentation measurements were conducted for each case.

## 3. Results and Discussion

**Figure 1:**
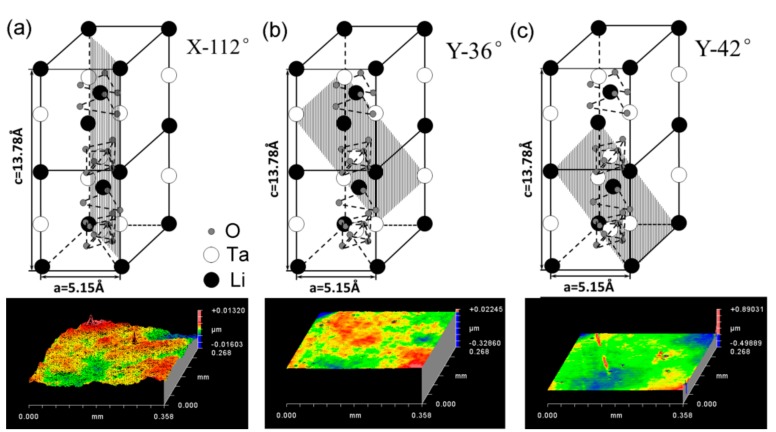
Schematic illustration of atomic arrangements for the typical orientations (**a**) X-112°, (**b**) Y-36° and (**c**) Y-42° in a LiTaO_3_ single crystal, and their surface morphologies by optical profile.

A LiTaO_3_ single crystal belongs to the trigonal *R*3*c* space group with an ion bonding structure and is commonly depicted by a hexagonal axes. Figure 1 shows a schematic illustration of the atomic arrangement in a LiTaO_3_ single crystal and the typical orientations ($1\bar{1}02$), ($\bar{1}012$) and ($01\bar{1}2$) i.e., X-112°, Y-36° and Y-42° are displayed in Figure 1a–c, respectively. The Li atom lies in an oxygen layer that is c/4 away from the Ta atom, and the Ta atom is centered between the oxygen layers. The representative surface morphologies of three planes are also exhibited in Figure 1 below the lattice diagrams. The surface roughness *R*a was 1.58 nm, 1.50 nm and 2.18 nm on the area of 360 μm × 270 μm for the X-112°, Y-36° and Y-42° planes, respectively. 

**Figure 2:**
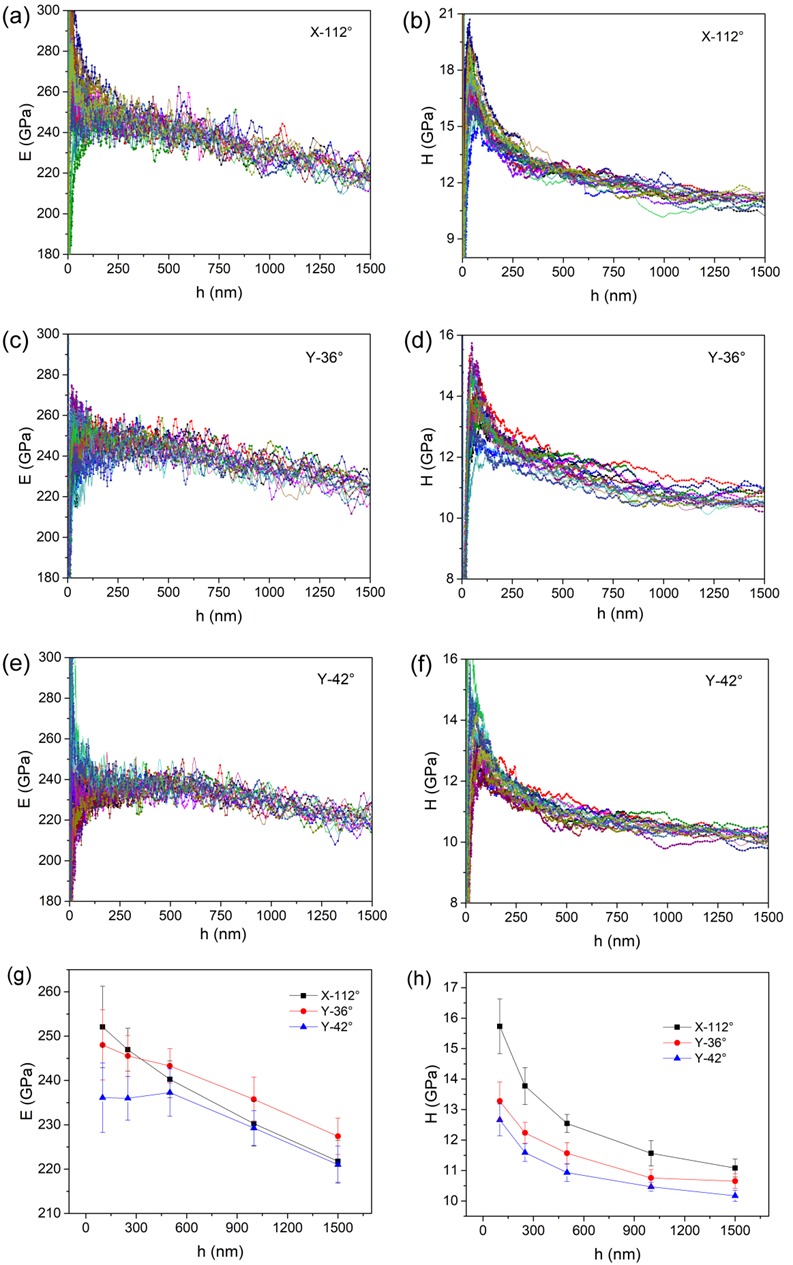
Elastic modulus and hardness as a function of displacement for (**a**,**b**) X-112°, (**c**,**d**) Y-36° and (**e**,**f**) Y-42° surfaces using the continuous stiffness module (CSM) method. The values of (**g**) the elastic modulus and (**h**) the hardness at 100 nm, 250 nm, 500 nm, 1000 nm and 1500 nm are listed for the three planes.

Figure 2 shows the elastic modulus *E* and hardness *H* as a function of pressed depth for three planes, in which eighteen tests were repeated in each case. The experimental results exhibited good reliability once the pressed depth was beyond about 100 nm. The disparities of the elastic modulus and hardness at a very shallow depth were mainly due to tip imperfections. Clearly, from the indentation size effect (ISE), it appeared that both *E* and *H* gradually decreased with increasing nanoindentation depth. The values of *E* and *H* at 100 nm, 250 nm, 500 nm, 1000 nm and 1500 nm are persented in Figure 2g,h. Obviously, ISE was more pronounced in the X-112° plane than the other planes. The mean value of *E* and *H* decreased from 252 GPa to 222 GPa and 15.7 GPa to 11.1 GPa for the X-112° plane, as the pressed depth increased from 100 nm to 1500 nm. The total range of *E* and *H* was about 248 GPa to 227 GPa and 13.3 GPa to 10.6 GPa for the Y-36° plane, 236 GPa to 221 GPa and 12.6 GPa to 10.2 GPa for the Y-42° plane, respectively. The X-112° plane exhibited the strongest resistance to plastic deformation, i.e., the highest hardness. The Y-42° plane was the weakest orientation in a single crystal of LiTaO_3_. It is worth mentioning that the *H* and *E* in LiTaO_3_ were comparable with He et al.’s result using the Berkovich indenter [15] and higher than Anasori et al.’s result using spherical tips [16].

The elastic modulus is an intrinsic mechanical parameter, which is tightly tied to atomic bonding. Generally, the elastic modulus is weakly dependent on testing conditions such as nanoindentation depth and strain rate. The investigation on the change of the elastic modulus is out of the scope of this work, which needs more details about the structural state along the depth. The ISE of hardness was commonly observed in metals and alloys, which is correlated with the plastic deformation mechanism [17]. Based on previous suggestions, a high density of dislocations at a shallow depth could strengthen the resistance to plastic deformation. While at deep nanoindentation, the dislocation density would decrease and the fully developed cracks might cause a softening effect on the hardness. 

**Figure 3:**
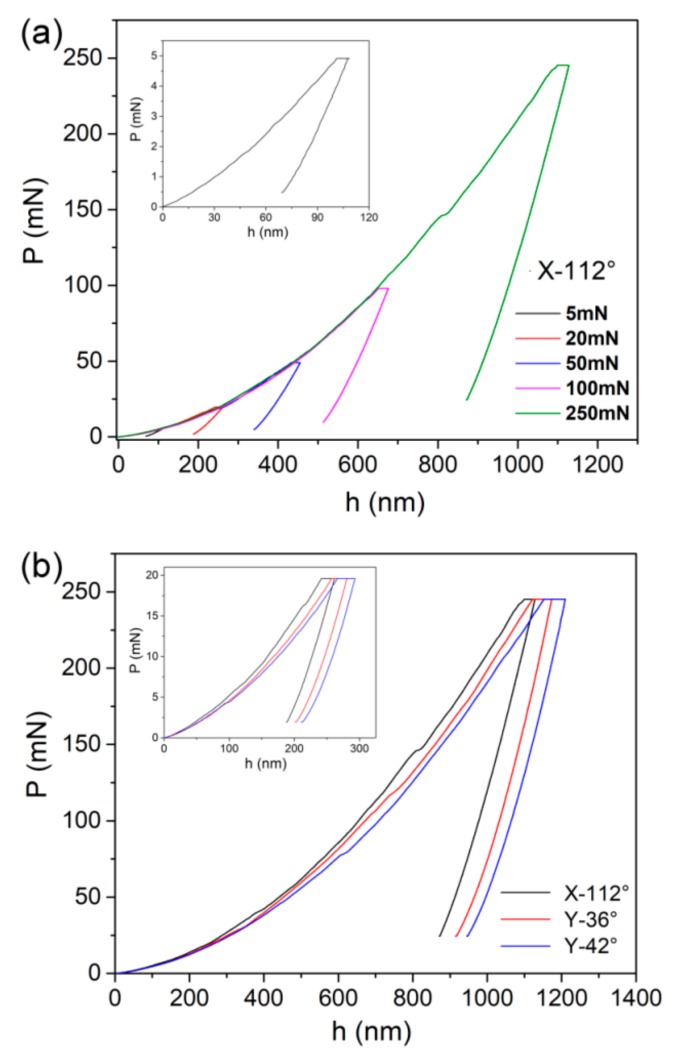
(**a**) The typical creep *P-h* curves at various holding depths for X-112° surface. The *P-h* curve at a shallow depth is enlarged in the inset. (**b**) Typical *P-h* curves of the three surfaces at 250 mN and 20 mN were compared.

Figure 3a shows the representative creep *P-h* (load versus displacement) curves for the X-112° plane at various peak loads, as an illustration. It was observed that the permanent deformation occurred on the holding stages, even for the 5 mN-holding test in the insets. Figure 3b shows the typical creep *P-h* curves at 50 mN (in the insets) and 250 mN for the three planes. Clearly, less displacement was required to attain the same load in the X-112° plane than the others, which was consistent with hardness result in Figure 2h.

**Figure 4:**
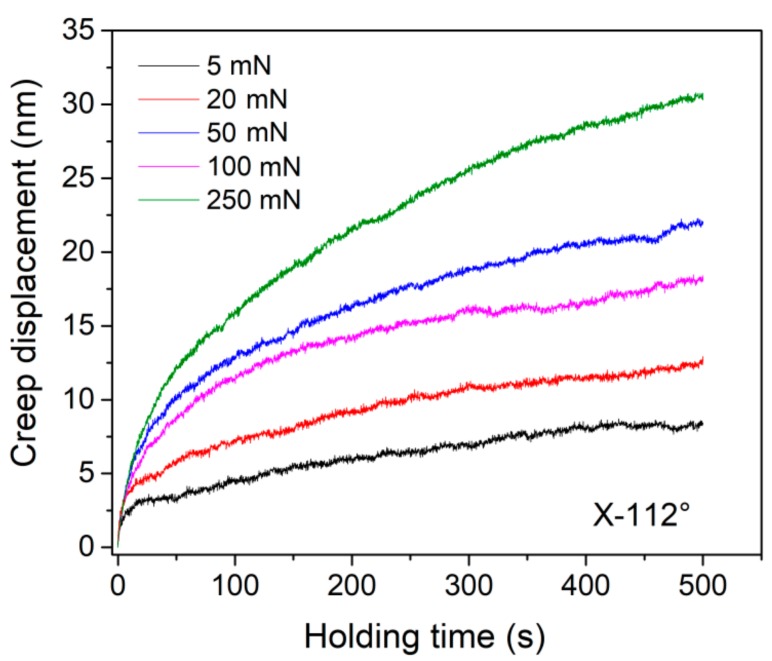
The representative creep flow curves at various holding depths for the X-112° surface.

Figure 4 shows the corresponding creep displacement during the holding stage at various peak loads for the X-112° plane, as an illustration. For a clear view, the onsets of creep flows were set to be zero in both time (X-axis) and displacement (Y-axis). Notwithstanding the holding time was quite limited, creep deformation was observed at room temperature in a LiTaO_3_ crystal, of which the melting point reached 1250 °C. This result could be mainly due to the great holding strain, which was estimated as 7.1% beneath the Berkovich indenter. Before the holding stage, severe plastic deformation had already occurred and the holding strain was far beyond the elastic limit, i.e., over-yielding holding. According to the creep feature in the conventional holding method, high stress and/or holding strain effectively promote the creep deformation. To be different from the conventional creep curves, only the first two stages (i.e., transient stage and steady-state stage) could be observed in nanoindentation creep flow. At the transient stage, creep displacement was precipitously increased in a short time while the creep rate dropped fast. Qualitatively, the duration of the transient stage was enlarged with an increasing holding load. At the steady-state stage, creep displacement almost linearly increased with holding time. In addition, the slope of the steady-state creep curve (i.e., the creep rate) gradually increased with the holding load. It should be noted that the creep deformation could be regarded as homogeneous and that no abrupt jump of displacement was detected during creep flow [18].

**Figure 5:**
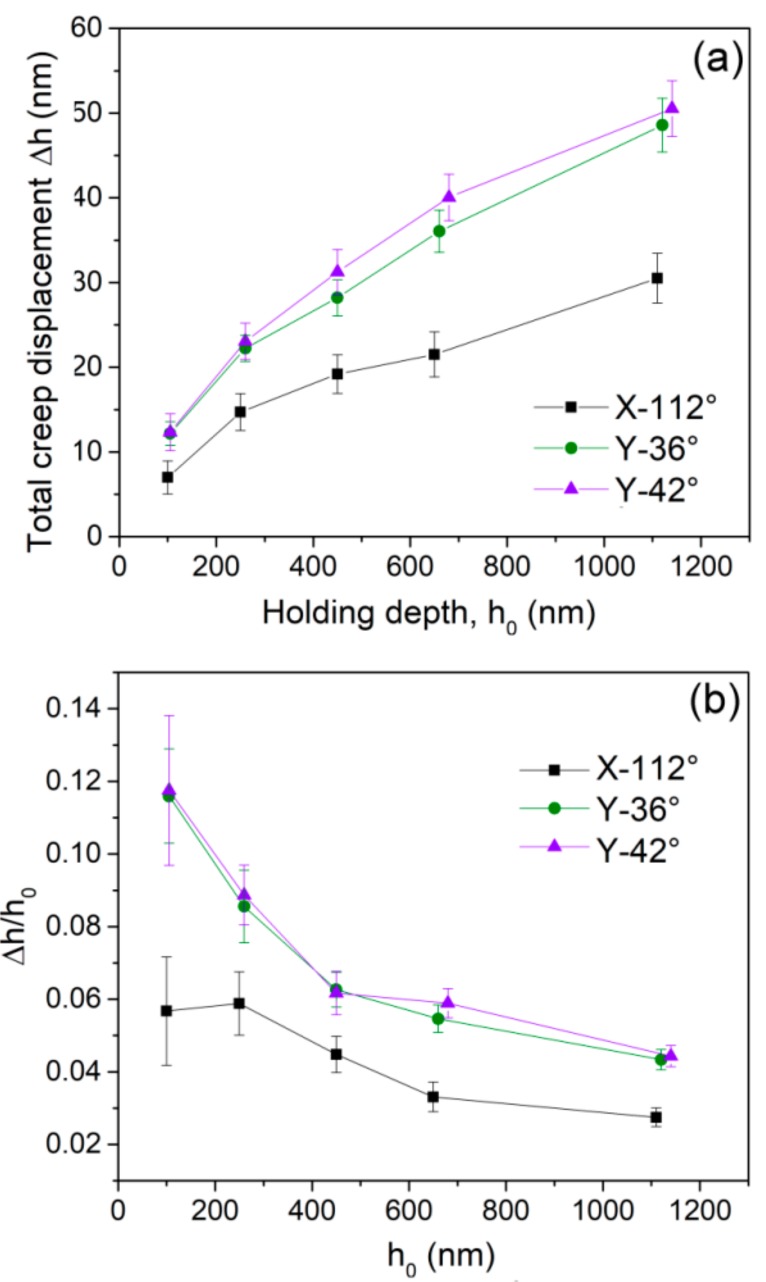
(**a**) Total creep displacements and (**b**) creep strains are depicted as a function of the initial holding depth.

The total creep displacements at the end of the holding stage, at various peak loads, were recorded for the three planes, as exhibited in Figure 5a. For self-similar Berkovich nanoindentation, the deformation region was in direct proportion to the pressed depth and the deformation strain was constant. Provided that creep resistance of a LiTaO_3_ crystal was size-independent, higher values of creep displacement were expected under larger holding loads. Evidently, the X-112° plane had the strongest creep resistance in the three orientations. Creep deformations in the Y-36° and Y-42° planes were similar and occurred relatively easily. The orientation effect on creep deformation perfectly conformed to the hardness features of the three planes in Figure 2h. As it was aforementioned that holding region beneath indenter plays an important role in creep displacement, creep strain could be adopted to study the nanoindentation size effect on creep behaviors of a LiTaO_3_ crystal. Here we defined creep strain as the total creep displacement versus the initial holding depth Δ*h*/*h*_0_. As it isshown in Figure 5b, creep strains at various holding depths were estimated for the three planes. 

In this scene, the creep strains nearly overlapped for the Y-36° and Y-42° planes. What is more, creep strain greatly decreased with the holding depth initially and then tended to be stable at deep nanoindentation for all the planes. Ideally, creep strain would be constant at the fixed holding strain and temperature. The herein the results indicate that creep resistance increased with increasing holding depth in LiTaO_3_, even though the holding strain was unchanged. Such indentation size effect on creep deformation is a conflict to that on hardness. From the perspective of the creep mechanism, indentation creep deformation at low temperature relied on dislocation glide [10]. Qualitatively, creep strain would be facilitated by the high density of the existing dislocation and low density of the obstacle such as the precipitates beneath the indenter. Briefly, creep flow mainly depended on the structural state at the beginning of the holding stage, whilst hardness relied more on the dynamic change of the structural state during nanoindentation. Thus, fully developed cracks at deep nanoindentation would cause “softened strength” by suddenly increasing the contact area beneath the indenter. It could be conceived that the density of plastic features such as dislocations and cracks during nanoindentation decreased with increasing pressed depth, as well as the density of defects introduced by polishing at surface and sub-surface. Hence the stronger creep resistance at deep nanoindentation could be explained. 

**Figure 6:**
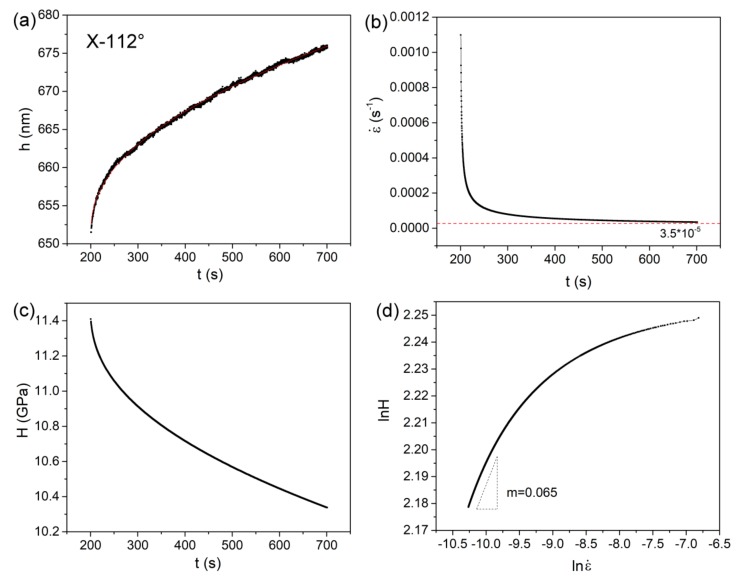
(**a**) Experimental creep deformation of X-112° at 100 mN holding was perfectly fitted by an empirical law; (**b**) change of strain rate during the holding stage; (**c**) change of hardness during the holding stage; (**d**) the log-log correlation between strain rate and hardness, the SRS value could be estimated by linear fitting on the steady-state creep part.

The present creep feature under nanoindentation was close to conventional creep behavior. Hence it has merits to estimate the strain rate sensitivity (SRS) [19,20] in order to reveal the creep mechanisms of three planes and their correlation with nanoindentation length scale. Here we selected a 100 mN-holding test in the X-112° plane, as an illustration, to calculate SRS. As shown in Figure 6a, the creep curve was perfectly fitted (R^2^ > 0.99) by an empirical law [21]:

*h*(*t*) = *h*_0_ + *a*(*t* − *t*_0_)^*b*^ + *kt* (1)

where *h_0_, t_0_* are the displacement and time at the beginning of the holding stage. *a*, *b*, *k* are the fitting constants. The value of SRS exponent *m* can be evaluated via:

(2) $$m=\frac{\partial \ln H}{\partial \ln\dot{\varepsilon }}.$$

In a standard Berkovich nanoindentation process, the strain rate during the holding stage can be calculated as:

(3) $$\dot{\varepsilon }=\frac{1}{h_{c}}\frac{dh_{c}}{dt}.$$

Nanoindentation hardness is defined as the applied load versus contact area:

(4) $$H=\frac{P}{Ch_{c}^{2}}.$$

*C* is the tip area coefficient and was rectified upon testing on standard fused silica, equal to 24.3 here. The contact displacement *h*_c_ could be obtained as $h_{c}=h-0.72\times P/S$. *S* is the stiffness and linearly increases with pressed depth. In the current study, it was unrealistic to detect *S* at each depth during the holding stage. In addition, the disparity of *S* at the beginning and end of the holding stage was no more than 5% for each sample. Besides, SRS was detected in the steady-state creep, wherein *S* was much close to that at the end of the holding stage. For simplicity, the *S* obtained from the unloading sequence of creep *P*-*h* curve was adopted to calculate hardness and strain rate. Figure 6b,c shows the changes in strain rate and hardness during the holding stage, which were deduced from the fitting line of creep curve, respectively. The strain rate rapidly decreased from 10^−3^ s^−1^ to 10^−5^ s^−1^ in the transient stage and tended to be stable about 3.5 × 10^−5^ s^−1^ in the steady-state stage. As it was indicated that strain rate under nanoindentation was one order of magnitude lower than that in conventional tension [22]. Thus, the localized brittle deformation could be suppressed under low strain rate (10^−4^ s^−1^–10^−6^ s^−1^) during creep flow. Hardness was decreased from 11.4 GPa to 10.3 GPa after 500 s holding. It should be noted that hardness at the beginning of the holding stage was consistent with the CSM result. Figure 6d shows the Logar-Logar correlation between the hardness and strain rate during the holding stage. Then, SRS can be obtained by linearly fitting on the part of steady-state creep. 

**Figure 7:**
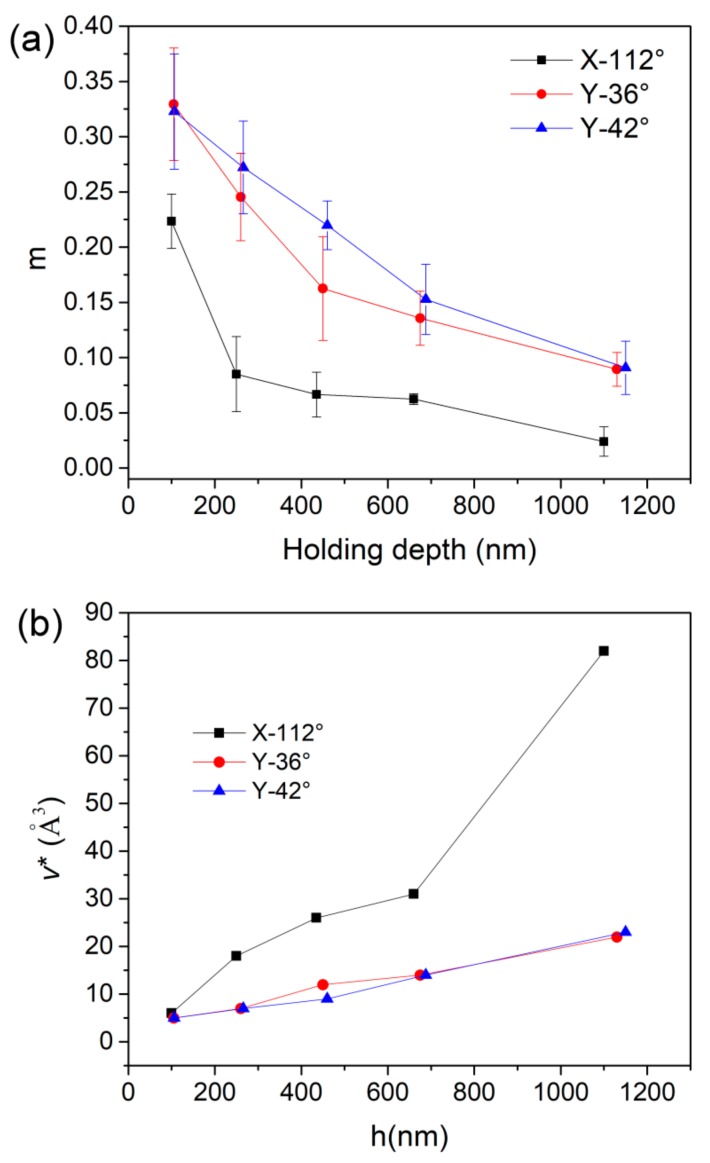
(**a**) The estimated values of SRS and (**b**) the activation volumes of dislocation nucleation at various holding depths for three planes.

The values of SRS at various holding depths were all computed for the three planes, as summarized in Table 1. As the holding depth increased from about 100 nm to 1100 nm, *m* decreased from 0.33 to 0.09 for the Y-36° and Y-42° planes, and 0.22 to 0.024 for the X-112° plane. The nanoindentation size effect on SRS could be more clearly recognized from the correlation between *m* and holding depth in Figure 7a. Such reduction of *m* on the holding depth has been reported in crystalline and amorphous solids [23,24,25]. In previous work, *m* decreased at an initial shallow depth and quickly tended to be stable once the pressed depth was beyond 100nm in alloys. In the present work, the reduction tendency of *m* was observed throughout a wide range from 100nm to 1100nm. In comparison, the values of SRS in the Y-36° and Y-42° planes were very close and evidently larger than the X-112° plane. The value of SRS *m* or stress exponent *n* (*n* = 1/*m*) was widely used as an indication of the creep mechanism at a high temperature [26]. For crystalline alloys or metals, dislocation move is dominating in creep flow as *m* falls in the range of 0.1 to 0.3. The detected values of *m* (0.33–0.09) in the Y-36° and Y-42° planes were comparable to the result by conventional creep testing under a high temperature and thousands of hours. This could explain that high stress and plastic deformation under nanoindentation dually stimulate the creep flow, equivalent to the thermal effect on atomic motion. For plastic holding, both stress level and atomic surroundings beneath indenter could meet the requirement of dislocation activation. While for the X-112° plane, the low *m* at deep nanoindentation could be due to unexpected obstructions for dislocation move. In addition, the low *m* in the magnitude of 10^-2^ has been revealed in nanoindentation creep of a nano-crystalline film, which was several times lower than that in conventional high-temperature creep [27].

According to Wei et al.’s work [28], the activation volume v* for dislocation nucleation could be expressed as:

(5) $$v^{*}=\frac{k_{B}T}{m\tau_{y}}.$$

Here *τ_y_* is the critical shear stress upon the traditional tensile or compressive tests, has an empirical correlation as *τ_y_* ≈ *H*/3√3. *k*_B_ is Boltzmann’s constant and *T* is the testing temperature. Once *H* and *m* were determined, the activation volume could be concomitantly obtained and summarized in Table 1.

Here hardness values upon CSM at various depths were adopted. Figure 7b exhibits the correlation between activation volume v* and holding depth for three planes. The values of v* in the Y-36° and Y-42° planes were nearly the same and gradually increased from 5 Å^3^ to 23 Å^3^, as the holding depth increased from about 100 nm to 1150 nm. In the meanwhile, the activation volume increased from 6 Å^3^ to 83 Å^3^ for the X-112° plane. Generally, the estimated activation volumes of dislocation nucleation in a LiTaO_3_ single crystal were inconsistent with the previously reported range for metals and alloys [29,30,31]. The activation volumes of the Y-36° and Y-42° planes were at the magnitude of the volume of a single atom, which seemed independent of the crystal structure. The point-like defects such as vacancies and impurities could be assumed to be the sources of dislocation nucleation. It is reasonable that structural defects were easily generated on the surface of LiTaO_3_ by polishing. For the X-112° plane, activation volumes around 30 Å^3^ were substantially comparative to the size of a vacancy. This is to say, dislocation was initiated at point-defect sites in the X-112° plane within a nanoindentation depth of about 750 nm. While at about 1100 nm, the activation volume was significantly enlarged to 83 Å^3^, which attains the magnitude of several atoms. Thus, cooperative migration of several atoms, i.e., the atomic cluster could be the mechanism of dislocation activation in the X-112° plane. The transition of dislocation activation mechanism suggests that nanoindentation was beyond the thickness of the damaged layer in the X-112° plane. It is reasonable that the damaged layer would be thinner in the X-112° plane than the other planes, due to its relatively higher elastic modulus and hardness. Basically, the larger the activation volume, the higher the activation energy that is required for dislocation nucleation. Therefore, it could be concluded that plastic initiation is more difficult to occur in the X-112° plane than the Y-36° and Y-42° planes. In addition, yielding a higher stress could be conceived in the X-112° plane. What is more, the stronger creep resistance in the X-112° plane than the others could also be explained upon the lager activation volume of dislocation nucleation. 

It should be noted that the activation volume for plastic initiation is based on mean-field theory and is commonly assumed as a constant for a certain composition. Thus, the nanoindentation size effect on the activation volume has rarely been studied upon the statistical method on yield stress [32] and rate-jump method on strain rate sensitivity [33]. In fact, the strong size effects on plastic deformation and mechanical properties have been revealed in numerous crystal and non-crystal materials [34,35,36]. The plastic morphologies could be greatly changed by reducing the sample size, even combined with the deformation mode transition. Therefore it has merit to study the size effect on activation volume of the plastic mechanism. In previous reports, both the sample size and nanoindentation size effects on the critical volume of the plastic unit, i.e., shear transformation zone in metallic glass have been investigated [37,38]. For a brittle LiTaO_3_ single crystal, the intrinsic mechanism of nanoindentation size effect on the thermal-assistant dislocation nucleation would be distinct to poly-crystal alloys and amorphous alloys. We can assume that the micro/nano-scale layer, damaged by polishing, plays an important role in the structural heterogeneity along the depth direction. Therefore the gradient changes of creep resistance and activation volume of dislocation nucleation could be explained. In the present work, the creep heterogeneity due to different structural orientations was revealed. To author’s best knowledge, it is the first report about activation volumes and their correlation with nanoindentation length scale in a brittle single crystal.

## 4. Conclusions

In summary, nanoindentation creep behaviors in the three typical orientations of a LiTaO_3_ single crystal were investigated upon a Berkovich indenter. The recorded total creep displacements were increased with the holding depth while creep strains were gradually decreased for all the planes. Creep behaviors in the Y-36° and Y-42° planes were much similar and distinct to those in the X-112° plane. Creep deformation was much more pronounced in the Y-36° and Y-42° planes than the X-112° plane, which was in accordant with the result that the X-112° plane owns the highest hardness. Based on the strain rate sensitivity of steady-state creep, the activation volume of dislocation nucleation was estimated in the range of 5Å^3^ to 23Å^3^ for the Y-36° and Y-42° planes and 6Å^3^ to 83Å^3^ for the X-112° plane. It is indicated that point-like defects would be the main sources of plastic initiation in a LiTaO_3_ single crystal, whilst cooperative migration of several atoms would be the mechanism of dislocation activation at deep places for the X-112° plane.

## Figures and Tables

**Table 1:** The values of *m* and v* deduced from steady-state creep for three cleavage planes.

	5 mN		20 mN		50 mN		100 mN		250 mN	
Orientation	*m*	v*	*m*	v*	*m*	v*	*m*	v*	*m*	v*
X-112°	0.22	6 Å^3^	0.085	18 Å^3^	0.067	26 Å^3^	0.062	31 Å^3^	0.024	83 Å^3^
Y-36°	0.33	5 Å^3^	0.25	7 Å^3^	0.16	12 Å^3^	0.14	14 Å^3^	0.089	22 Å^3^
Y-42°	0.32	5 Å^3^	0.27	7 Å^3^	0.22	9 Å^3^	0.15	14 Å^3^	0.091	23 Å^3^
